# Short-term radiographic changes of extruded NeoSealer Flo in a simulated enlarged-apex model: influence of moisture and periapical support

**DOI:** 10.1007/s00784-026-07175-1

**Published:** 2026-10-02

**Authors:** Andrea Spinelli, Valentina Pergola, Carlo Prati, Maria Giovanna Gandolfi, Fausto Zamparini

**Affiliations:** 1 Department of Biomedical and Neuromotor Sciences, Endodontic Clinical Section, Dental School, University of Bologna, Bologna, Italy; 2 Department of Biomedical and Neuromotor Sciences, Laboratory of Green Biomaterials and Oral Pathology, Dental School, University of Bologna, Bologna, Italy

**Keywords:** Apical extrusion, Bioceramic sealer, Calcium silicate, Endodontics, Periapical radiography, Root canal obturation

## Abstract

**Objectives:**

To radiographically evaluate short-term changes in NeoSealer Flo extruded beyond enlarged apical preparations under different moisture and apical-support conditions.

**Materials and methods:**

Thirty-two extracted single-rooted human teeth were randomly allocated to four experimental conditions (*n* = 8 per group). Teeth were prepared with reciprocating NiTi instruments to size 50/0.05 and filled with a matched gutta-percha cone and a premixed bioceramic sealer. The conditions combined the presence or absence of sponge support around the apex with dry or phosphate-buffered saline PBS-moistened canals. Standardized radiographs were acquired immediately after filling (T0), at 7 days (T1), and at 28 days (T2). Intracanal radiopaque area (Area of the canal, AOC) and apically extruded radiopaque area (Area of Extrusion, AE) were traced within a calibrated 6 × 10-mm region of interest. Separate random-intercept linear mixed models included group, time, and their interaction; pairwise comparisons used Sidak adjustment.

**Results:**

Mean AOC changed by no more than 0.56 mm² within groups. The group-by-time interaction was not significant for AOC (Wald χ²(6) = 5.31, *p* = 0.505) but was significant for AE (Wald χ²(6) = 21.72, *p* = 0.001). From T0 to T2, mean AE changed by + 3.38 mm² (Group 1; dry/sponge), + 0.25 mm² (Group 2; moist/sponge), + 1.50 mm² (Group 3; dry/no sponge), and + 0.41 mm² (Group 4; moist/no sponge).

**Conclusions:**

Intracanal radiopaque area remained relatively stable over 28 days, whereas the extruded radiopaque area differed among experimental conditions. The largest mean increase occurred in the dry, sponge-supported group.

**Clinical relevance:**

Local moisture and periapical status may influence the radiopaque area of extruded NeoSealer Flo. However, the radiographic changes do not directly demonstrate material expansion, dissolution, resorption, or biological integration. Apical extrusion should be minimized in clinical practice.

## Introduction

 Hydraulic calcium silicate-based root canal sealers are frequently used with matched single cone obturation [1, 2]. Premixed calcium silicate sealers, often marketed as “bioceramic” sealers, are widely used because of their ready-to-use delivery, flow, alkalizing activity, calcium release, and capacity to nucleate surface mineral deposits after immersion with phosphate-containing fluids [1–5]. Unlike conventional paste–paste sealers, these materials hydrate and set through interaction with water from dentin and tissue fluids; therefore, moisture availability may influence their setting and subsequent behavior [1, 4, 6–8].

NeoSealer Flo is a premixed bioceramic sealer containing tricalcium silicate (< 25%) and dicalcium silicate (< 10%) as bioactive components; calcium aluminate (< 25%), calcium aluminum oxide (grossite; < 6%), and tricalcium aluminate (< 5%); tantalite (50%) as a radiopacifier; and traces of calcium sulfate (< 1%) [4]. It is designed for use with both warm and cold obturation techniques. Recent clinical studies reported success rates above 85% over follow-up periods of up to 3 years, with sealer extrusion in 29%–48% of single cone cases [9, 10].

Apical control and moisture management are particularly important when the apical anatomy is enlarged or lacks a constriction. Clinical studies have described changes over time in the radiographic appearance of extruded premixed calcium silicate sealers [11, 12], calcium hydroxide-containing sealers [13], and zinc oxide–eugenol sealers [14].

Dimensional stability is a core sealer property, and conventional in vitro tests have documented material-dependent contraction or expansion [14]. Laboratory investigations have shown that foraminal enlargement can increase the risk of filling-material extrusion [15], whereas simulated immature teeth may be better managed with an apical plug than with a single cone technique when marginal adaptation is the primary outcome [16]. Clinical studies have reported heterogeneous fates for extruded sealers, ranging from persistent radiopacity to partial or complete radiographic disappearance [17–21].

Periapical radiography remains a first-line method for evaluating root canal treatment outcomes. When image acquisition and analysis are carefully standardized, serial radiographs can provide clinically meaningful evidence of changes in extruded material and may reasonably suggest that physical or biological events are occurring in the periapical area, such as variations in radiopacity, hydration-related changes, mineral precipitation, dissolution, resorption, or biological integration [21, 22].

Previous studies have investigated NeoSealer Flo using different obturation techniques, showing that filling characteristics may vary according to the technique used [23]. Moisture conditions during root canal obturation may also influence the behavior of calcium silicate-based sealers [24], while foraminal enlargement has been associated with an increased occurrence of apical sealer extrusion [25]. However, evidence specifically addressing the radiographic behavior of extruded NeoSealer Flo under enlarged apical preparations and contrasting moisture conditions remains limited, and the interaction between intracanal moisture and the local space available for extrusion has not been specifically characterized.

Therefore, this in vitro study evaluated short-term changes in the radiographically measured intracanal and apically extruded areas of NeoSealer Flo immediately after filling, at 7 days, and at 28 days. Four experimental conditions combined the presence or absence of sponge support around the apex with dry or PBS-moistened canals. The null hypotheses were that radiopaque area would not change over time and that temporal changes would not differ among the four conditions.

## Materials and methods

### Study design and reporting

**Fig. 1:**
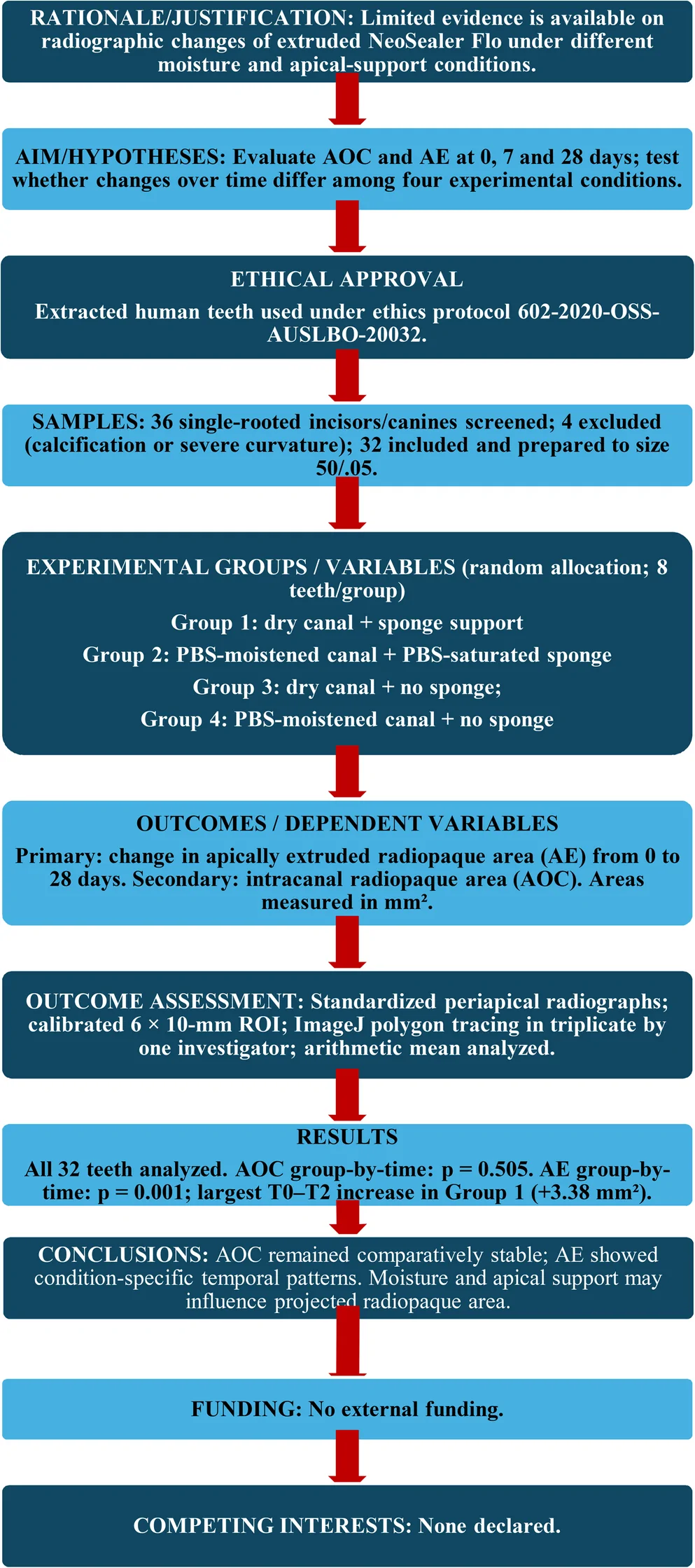
PRILE flowchart of the study

This study used extracted human teeth and was prepared with reference to the PRILE 2021 recommendations for reporting laboratory studies in Endodontology (Fig. 1) [26]. The experiment was performed in the Endodontic Clinical Section, University of Bologna, Italy.

### Sample-size calculation and primary outcome

The primary outcome was the change in apically extruded radiopaque area (AE) from baseline to 28 days. An a priori sample-size calculation used a two-group approximation based on a previously published in vitro sealer-extrusion model, in which a 33% between-condition difference in dimensional change with an estimated standard deviation of 20% was considered relevant [22]. This corresponded to a standardized effect size of 1.65. For a two-sided significance level of 0.05 and 80% power, a minimum of seven specimens per group was required. This was increased to eight specimens per group to account for potentially unusable specimens or radiographic measurements, resulting in a total sample of 32 teeth. Intracanal radiopaque area (Area of the Canal; AOC) was considered a secondary outcome.

### Specimen selection and root canal preparation

Teeth extracted for periodontal reasons were collected and used under ethics protocol 602-2020-OSS-AUSLBO-20,032.

Thirty-six extracted maxillary or mandibular single-rooted incisors and canines were screened radiographically. Four teeth were excluded because of canal calcification or severe curvature, leaving 32 specimens. Teeth were decoronated to achieve a standardized root length of 13 ± 1 mm and individually identified. Working length was determined by advancing a size 10 K-file until its tip became visible at the apical foramen. Coronal preflaring was performed with Gates–Glidden drills, followed by manual scouting with sizes 10, 15, and 20 K-files. Canals were then prepared using Reciproc Blue instruments (R25, R40, and R50; VDW, Munich, Germany), with the final preparation corresponding to size 50/0.05.

During preparation, each canal was irrigated with 5 mL of 5.25% sodium hypochlorite (Niclor 5, Ogna, Muggiò, Italy), delivered through a 27-gauge irrigation needle connected to a Luer-lock syringe. The needle tip was positioned 3 mm short of the working length. Instruments were cleaned using a sterile endodontic sponge after each insertion, and instrumentation continued until no visible debris remained on the instrument or in the irrigant. Final irrigation was performed with 1 mL saline. Instrumentation, irrigation, and obturation procedures were based on previous in vitro investigations [27, 28].

### Experimental apical conditions and group allocation

**Fig. 2:**
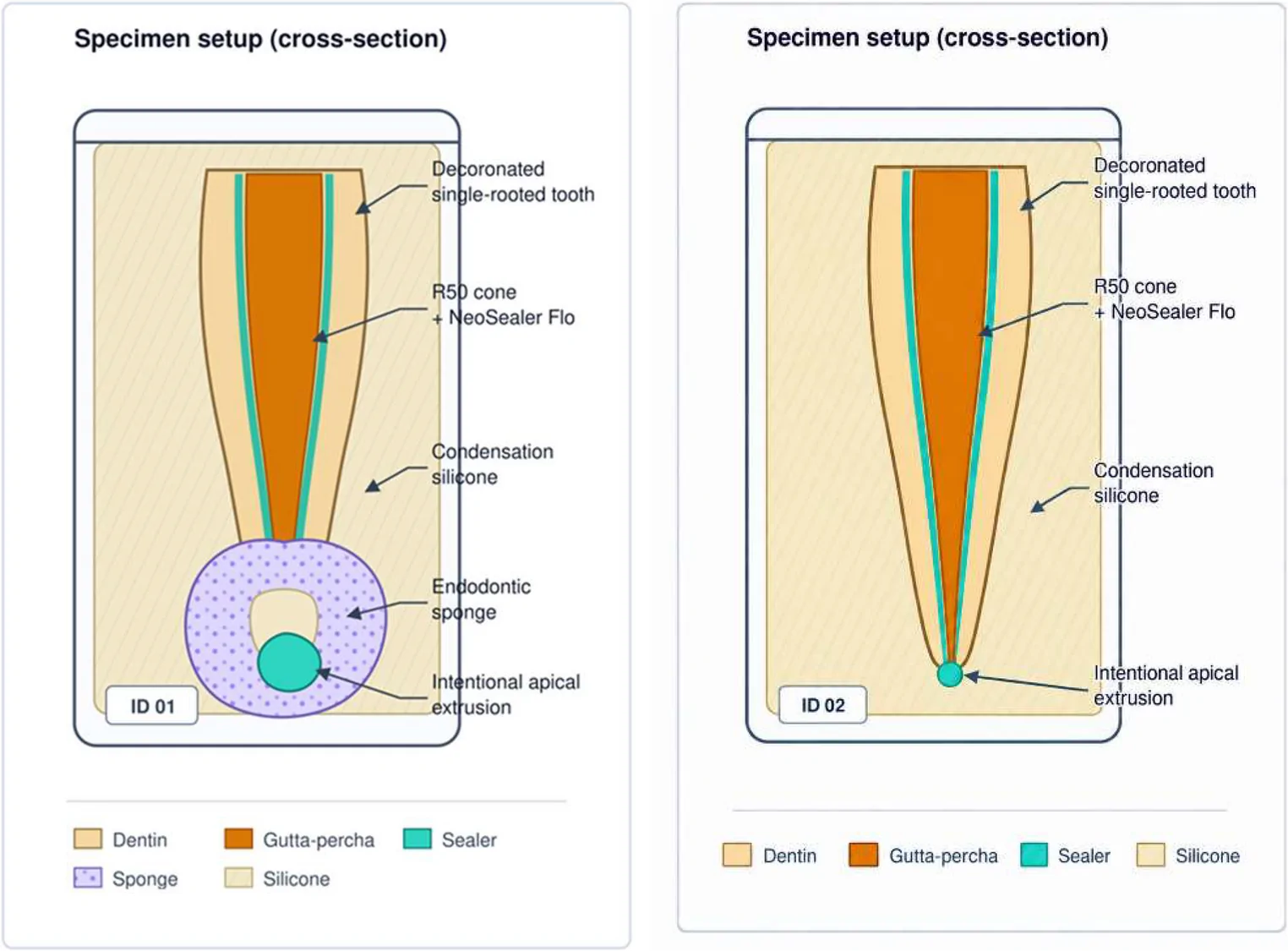
Experimental models used in the present study

The 32 specimens were randomly allocated to four groups (*n* = 8). Each root was mounted in a numbered plastic container filled with condensation silicone (Optosil Comfort Putty; Kulzer, Hanau, Germany). For the two sponge-supported conditions, an endodontic sponge was positioned around the root apex within the silicone to create a space for extruded material (Fig. 2). In the unsupported conditions, the root was placed directly in the silicone without a sponge with a residual space remained around the apex when the tooth was positioned within the artificial socket. The four experimental conditions are summarized in Table 1.

**Table 1:** Experimental conditions (*n* = 8 specimens per group)

Group	Apical support	Canal condition	Sponge condition	Experimental interpretation
1 (D-S)	Sponge present	Dry	Dry	Dry canal with apical support
2 (PBS-S)	Sponge present	PBS-moistened	PBS-moistened	Moist canal with apical support
3 (D-NS)	Sponge absent	Dry	Not applicable	Dry canal without apical support
4 (PBS-NS)	Sponge absent	PBS-moistened	Not applicable	Moist canal without apical support

Group 1: Dry/Sponge (D-S); Group 2: PBS/Sponge (PBS-S); Group 3: Dry/No Sponge (D-NS); Group 4: PBS/No Sponge (PBS-NS)

In Groups 2(PBS-S) and 4(PBS-NS), the canal was moistened with 3 mL PBS (pH 7.4 at room temperature) delivered through a 27-gauge needle; the sponge in Group 2 (PBS-S) was also saturated with PBS. PBS was used as simulated body fluid.

In Groups 1(D-S) and 3(D-NS), the canals were dried with paper points and no ethanol-based drying step was used. The sponge was an experimental support that created a reproducible compliant space around the apex; it was not intended as a histological model of a granuloma or abscess.

### Root canal filling

All root canals were filled by a single trained operator using NeoSealer Flo (Avalon Biomed, Houston, TX, USA) and a matched Reciproc R50 gutta-percha cone with a cold single cone technique. The gutta-percha cone was inserted to the previously determined working length, cut at the canal orifice, and covered with a temporary filling material (Coltosol F; Coltène/Whaledent AG, Altstätten, Switzerland) [29]. The specimens remained in their numbered artificial sockets throughout the experiment and were stored in an incubator at 37 °C and 100% relative humidity, maintained using a connected humidifier.

### Radiographic acquisition and image analysis

Standardized two-dimensional periapical radiographs were obtained immediately after filling (T0), at 7 days (T1), and at 28 days (T2). Phosphor plates measuring 3 × 4 cm were scanned with a VistaScan Mini View system (Dürr Dental, Bietigheim-Bissingen, Germany) at 1270 dpi. Exposure was performed with an X-Mind DC intraoral unit (Acteon, Mérignac, France) in digital mode at 70 kV, 8 mA, and 0.20 s. An alginate index reproduced the position of the specimen container and phosphor plate at each time-point. Measurements were performed in ImageJ (National Institutes of Health, Bethesda, MD, USA) by a single trained examiner, different from the operator who performed root canal filling procedures. The examiner was not blinded to group allocation because the presence or absence of the apical sponge support cannot be concealed during the assessment.

**Fig. 3:**
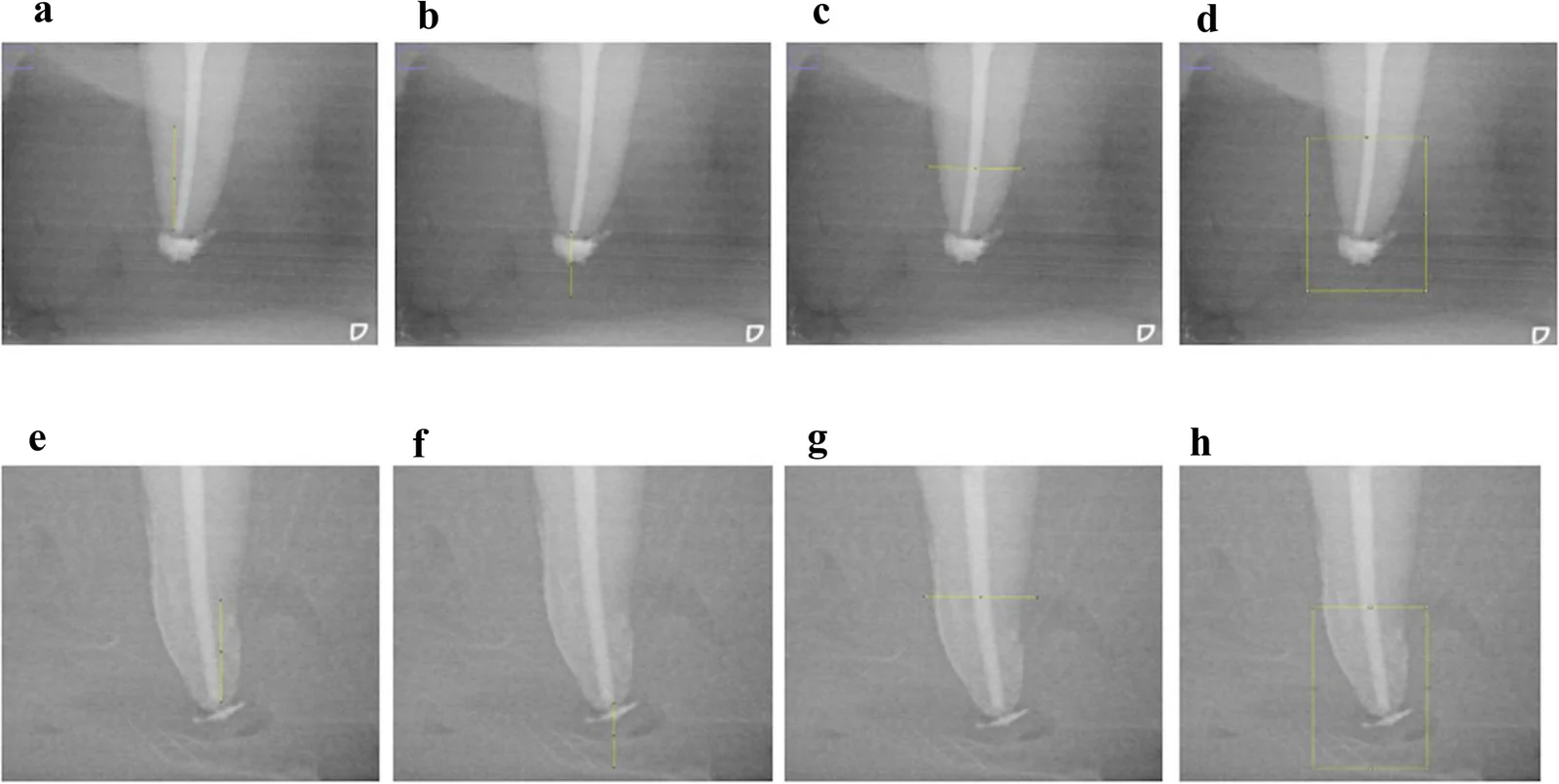
Two representative specimens of teeth with sponge (**a**-**d**) and without sponge (**e**-**h**). The ROI was constructed by measuring 6 mm into the canal (**a**, **e**), 4 mm from the apex (**b**, **f**), 6 mm width from the center of the canal (**c**, **g**). The final ROI is reported in yellow (**d**, **h**)

A 1-mm radiographic reference was used for calibration (39.4588 pixels/mm). A rectangular region of interest measuring 10 mm in the apicocoronal direction (6 mm coronal and 4 mm apical to the radiographic apex) and 6 mm mesiodistally was applied consistently, giving a total area of 60 mm² (Fig. 3). Polygon selections were traced around (1) the radiopaque filling area within the canal in the region of interest (Area of the Canal; AOC) and (2) the radiopaque material beyond the apex (Area of Extrusion; AE). Each area was measured three times, and the arithmetic mean was analyzed. 

### Statistical analysis

Statistical analyses were made with Stata Now 19.5 (Stata, College Station, TX, USA).

Continuous outcomes were summarized as mean ± standard deviation. Separate linear mixed-effects models were fitted for AOC and AE, with group, time, and the group-by-time interaction as fixed effects and specimen as a random intercept. Time was treated as categorical, and models were estimated by restricted maximum likelihood. Global fixed effects were assessed with Wald chi-square tests. Model-based pairwise contrasts and their standard errors were calculated from the estimated fixed effects and their covariance matrix, using a normal approximation. Unadjusted 95% confidence intervals were reported. For each outcome, Sidak adjustment was applied separately to the three temporal comparisons within each group and the six group comparisons at each time. All tests were two-sided, with statistical significance set at *p* < 0.05.

## Results

A total of 32 teeth were analyzed at T0, T1, and T2, with eight specimens in each experimental condition. Descriptive results are presented in Table 2.

**Table 2:** Radiographically measured areas by condition and time (mean ± standard deviation; mm²)

Group	Outcome	T0: after filling	T1: 7 days	T2: 28 days	Variation T2 − T0
1 (D-S)	AOC	4.35 ± 0.72	4.30 ± 0.54	4.81 ± 0.61	+ 0.46
	AE	4.52 ± 2.45	6.57 ± 3.53	7.90 ± 3.27	+ 3.38
2 (PBS-S)	AOC	4.16 ± 0.56	3.85 ± 0.94	4.41 ± 0.70	+ 0.25
	AE	7.50 ± 5.88	5.29 ± 4.39	7.75 ± 6.16	+ 0.25
3 (D-N)	AOC	5.35 ± 1.24	5.00 ± 1.25	5.41 ± 1.14	+ 0.06
	AE	8.88 ± 5.02	8.71 ± 5.63	10.38 ± 6.18	+ 1.50
4 (PBS-NS)	AOC	3.59 ± 0.41	3.50 ± 0.53	3.65 ± 0.48	+ 0.06
	AE	2.91 ± 1.38	3.10 ± 2.12	3.33 ± 2.53	+ 0.41

Area of the canal (AOC), Area of extrusion (AE)

### Area of the canal (AOC)

**Fig. 4:**
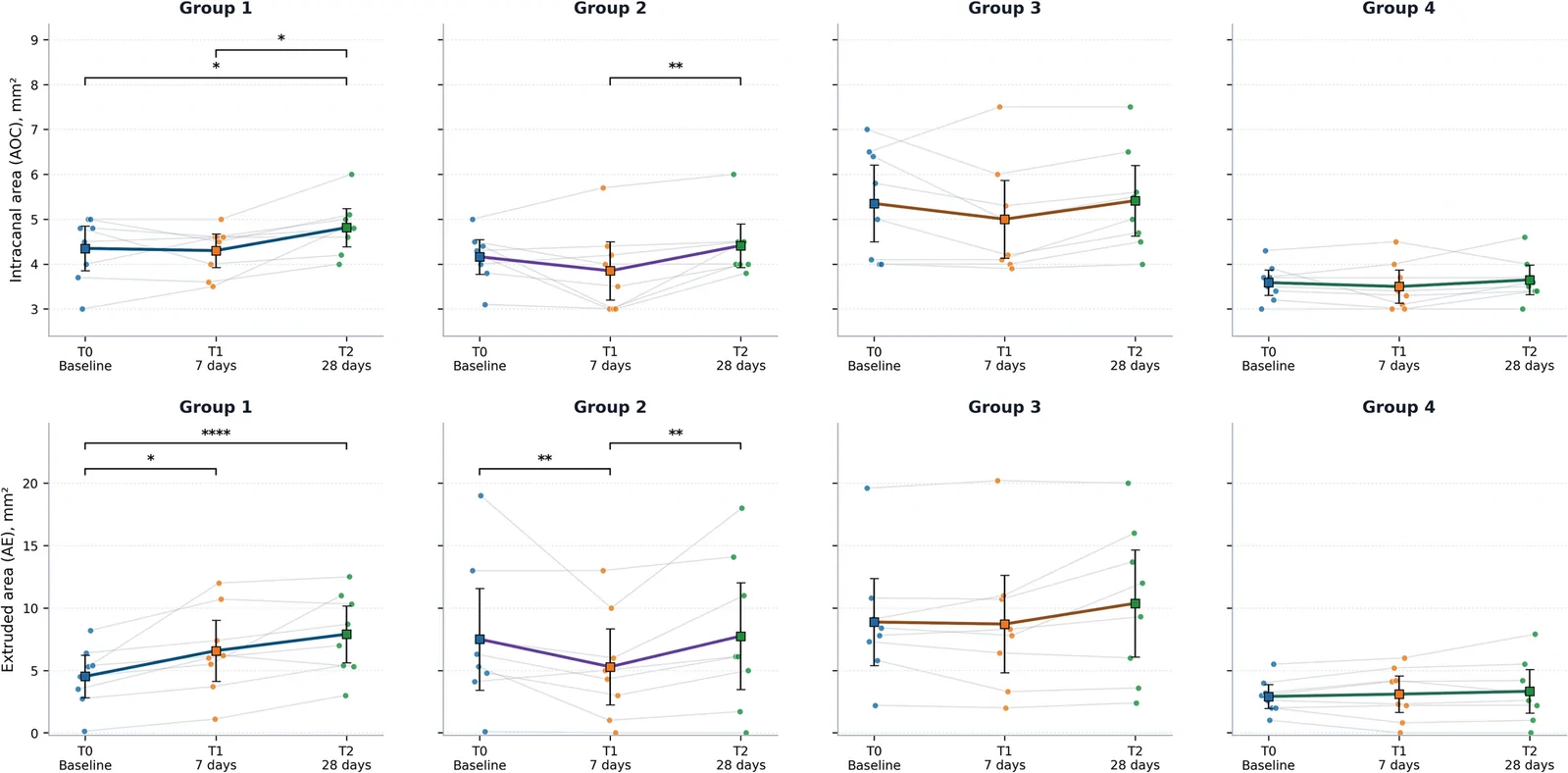
Specimen-level trajectories and group means for Area of the Canal (AOC) and Area of Extrusion (AE) radiopaque areas immediately after filling (T0), at 7 days (T1), and at 28 days (T2). Thin lines connect repeated measurements from the same specimen; squares and error bars show means with 95% normal-approximation confidence intervals. Brackets indicate significant Sidak-adjusted within-group contrasts (*p < 0.05, **p < 0.01, ****p < 0.0001)

Mean AOC showed limited short-term variation (Table 2; Fig. 4). From T0 to T2, the mean changes were + 0.46 mm² in Group 1 (D-S), + 0.25 mm² in Group 2 (PBS-S), + 0.06 mm² in Group 3 (D-NS), and + 0.06 mm² in Group 4 (PBS-NS). The largest mean difference between any two time-points within a group was 0.56 mm², observed in Group 2 (PBS-S) between T1 and T2.

### Area of extrusion (AE)

AE showed greater temporal and between-specimen variability than AOC. In Group 1 (D-S), mean AE increased from 4.52 ± 2.45 mm² at T0 to 7.90 ± 3.27 mm² at T2 (Table 2; Fig. 4). Group 2 (PBS-S) showed a decrease at T1 followed by a return to approximately baseline at T2, with means of 7.50 ± 5.88, 5.29 ± 4.39, and 7.75 ± 6.16 mm² at T0, T1, and T2, respectively. Group 3 (D-NS) had the highest mean AE at all time points and reached 10.38 ± 6.18 mm² at T2. Group 4 (PBS-NS) showed the lowest mean values throughout, increasing from 2.91 ± 1.38 mm² at T0 to 3.33 ± 2.53 mm² at T2. Standard deviations were large relative to the corresponding means in several group–time combinations, indicating substantial heterogeneity.

### Mixed-model and post hoc comparisons for AOC and AE

Figure 4 shows specimen-level trajectories and group means for AOC and AE. Table 3 summarizes the global mixed-model tests.

**Table 3:** Mixed-effects model fixed-effect tests

Outcome	Effect	Wald χ²	df	*p* value
AOC	Group	20.76	3	< 0.001
AOC	Time	19.02	2	< 0.001
AOC	Group × time	5.31	6	0.505
AE	Group	8.88	3	0.031
AE	Time	19.01	2	< 0.001
AE	Group × time	21.72	6	0.001

Separate random-intercept models were fitted for Area of the Canal (AOC) and Area of Extrusion (AE) by restricted maximum likelihood. Tests used Wald chi-square statistics with a normal approximation

For AOC, the mixed-effects model identified significant overall effects of group (Wald χ²(3) = 20.76, *p* < 0.001) and time (Wald χ²(2) = 19.02, *p* < 0.001), whereas the group-by-time interaction was not significant (Wald χ²(6) = 5.31, *p* = 0.505). Sidak-adjusted within-group contrasts showed small increases in Group 1 (D-S) from T0 to T2 (estimated difference 0.46 mm², 95% CI 0.09 to 0.83; adjusted *p* = 0.041) and from T1 to T2 (0.51 mm², 95% CI 0.14 to 0.88; adjusted *p* = 0.019). In Group 2 (PBS-S), AOC increased from T1 to T2 (0.56 mm², 95% CI 0.19 to 0.93; adjusted *p* = 0.008). No adjusted time-related contrast was significant in Groups 3 (D-NS) or Group 4 (PBS-NS).

Sidak-adjusted between-group contrasts for AOC showed higher values in Groups 3 (D-NS) than in Group 2 (PBS-S) and Group 4 (PBS-NS) at T0 (estimated differences 1.19 mm², 95% CI 0.39 to 1.98, adjusted *p* = 0.021; and 1.76 mm², 95% CI 0.97 to 2.56, adjusted *p* < 0.001, respectively) and at T1 (1.15 mm², 95% CI 0.35 to 1.95, adjusted *p* = 0.028; and 1.50 mm², 95% CI 0.70 to 2.30, adjusted *p* = 0.001, respectively). At T2, AOC was higher in Group 1 (D-S) than in Group 4 (PBS-NS) (1.16 mm², 95% CI 0.37 to 1.96; adjusted *p* = 0.025) and higher in Group 3 (D-NS) than in Group 4 (PBS-NS) (1.76 mm², 95% CI 0.97 to 2.56; adjusted *p* < 0.001). No other adjusted between-group contrast was statistically significant.

For AE, significant effects of group (Wald χ²(3) = 8.88, *p* = 0.031), time (Wald χ²(2) = 19.01, *p* < 0.001), and the group-by-time interaction (Wald χ²(6) = 21.72, *p* = 0.001) were detected, indicating that temporal changes differed among experimental groups (Table 3). In Group 1(D-S), AE increased from T0 to T1 (estimated difference 2.05 mm², 95% CI 0.60 to 3.51; adjusted *p* = 0.017) and from T0 to T2 (3.38 mm², 95% CI 1.92 to 4.83; adjusted *p* < 0.001); the T1-to-T2 contrast was not significant. In Group 2 (PBS-S), AE decreased from T0 to T1 (− 2.21 mm², 95% CI − 3.67 to − 0.76; adjusted *p* = 0.009) and increased from T1 to T2 (2.46 mm², 95% CI 1.01 to 3.92; adjusted *p* = 0.003), with no significant T0-to-T2 difference. No adjusted temporal contrast was significant in Group 3 (D-NS) or Group 4 (PBS-NS).

Sidak-adjusted between-group contrasts for AE showed higher values in Group 3 (D-NS) than in Group 4 (PBS-NS) at T0 (estimated difference 5.96 mm², 95% CI 1.68 to 10.24; adjusted *p* = 0.037) and at T2 (7.05 mm², 95% CI 2.77 to 11.33; adjusted *p* = 0.007). No adjusted between-group contrast was significant at T1, and no other contrast reached statistical significance.

## Discussion

This study evaluated the short-term radiographic behavior of NeoSealer Flo after apical extrusion under four experimental conditions. Intracanal radiopaque area remained relatively stable over 28 days, whereas AE followed different temporal patterns across conditions. The significant group-by-time interaction for AE supports rejection of the corresponding null hypothesis. However, AE already differed between Groups 3 (D-NS) and 4 (PBS-NS) at baseline, and variability was substantial; between-group differences in temporal trajectories should therefore be interpreted cautiously.

The experimental design was motivated by heterogeneity in clinical protocols for premixed calcium silicate sealers, particularly regarding obturation technique and moisture control [30]. Methodological reviews have also emphasized that hydraulic sealers require testing protocols adapted to their water-mediated setting reaction rather than the direct application of methods developed for conventional paste–paste materials [31, 32]. Accordingly, the four conditions varied intracanal moisture and the presence of compliant support around the apical foramen.

The clearest divergent trends were observed in the sponge-supported groups. In the dry, sponge-supported group, mean AE progressively increased from baseline, whereas in the moist, sponge-supported group it decreased at 7 days and returned toward baseline at 28 days. These findings suggest that the space available for material redistribution and local moisture may influence the projected radiopaque area of the extruded sealer.

Canals assigned to the dry condition were dried with paper points only, and no ethanol-based drying step was used. This provided a clinically applicable drying protocol while avoiding chemical desiccation. Previous work has shown that dentin moisture conditions can affect sealer adhesion [33]. Moisture conditions and environmental pH have been shown to influence the setting time and microhardness of premixed calcium silicate-based root canal sealers [34].

Several physicochemical processes could contribute to the observed changes. NeoSealer Flo contains reactive calcium silicates, (i.e. tricalcium silicate). Previous in vitro characterization reported relatively high open-pore volume, water absorption (33.0 ± 11.0%), and solubility (7.10 ± 3.5%) [4]. The relatively high open porosity and water uptake reported for NeoSealer Flo are also consistent with the material-dependent differences in porosity, fluid uptake, and water sorption previously described for other hydraulic calcium silicate cements, including MTA and Biodentine [35, 36]. An interconnected pore network could facilitate water penetration, hydration of reactive phases, and calcium-ion release. In the dry condition, delayed or incomplete hydration might prolong setting and allow continued redistribution of freshly extruded material. Conversely, early water uptake and leaching in the PBS-moistened condition might contribute to the initial decrease in radiopaque area. After aging in Hank’s balanced salt solution, ESEM-EDX and vibrational analyses documented a calcium- and carbon-rich surface layer on NeoSealer Flo, predominantly containing calcium carbonate phases such as calcite, together with limited calcium-phosphate/apatite nucleation [4, 5].

PBS was used to simulate selected physicochemical characteristics of body fluids, providing an aqueous saline medium with buffering capacity at physiological pH. Although temperature, humidity, and canal wetness were controlled to approximate clinical conditions, laboratory setting times may differ from those in vivo [7]. Furthermore, the sponge should be regarded only as an experimental support and not as a histological surrogate for a granuloma, cyst, or abscess. In the groups without the sponge, a residual space remained around the apex when the tooth was positioned within the artificial socket. Extruded sealer could fill this space and potentially contact the surrounding silicone, which may have influenced the extent of sealer extrusion.

The findings may be relevant to enlarged apical anatomy, in which control of sealer placement is difficult. Foraminal enlargement has been associated with increased extrusion [15, 37], and an apical plug can provide better marginal adaptation than a customized single cone with a calcium silicate sealer in simulated immature teeth [16]. Clinical protocols have also used apical plugs in teeth with necrotic pulps and immature apices [38]. Although several clinical studies found that sealer extrusion did not necessarily preclude healing [17–20, 39], outcomes depend on material composition, anatomy, microbial control, filling quality, and the preoperative periapical condition. Recent long-term observations with NeoSealer Flo reported radiographic modification in only a minority of extruded cases [10], reinforcing that radiographic disappearance is neither uniform nor a prerequisite for healing [21]. Clinical evidence further suggests that the radiographic fate of extruded sealers may be material-dependent and should not necessarily be interpreted as a surrogate for treatment failure. Kangseng et al. reported comparable clinical success between calcium silicate- and epoxy resin-based sealers, with no significant difference in extrusion rates and only occasional partial or complete disappearance of the extruded material [40]. Similarly, a recent 5-year cohort study using a calcium silicate-based sealer found that extrusion was not associated with poorer treatment outcome, while both partial and complete radiographic resorption were observed during follow-up [41]. In a randomized clinical trial comparing a bioceramic-containing sealer with an epoxy resin-based sealer using the same carrier-based technique, high 12-month healing rates were observed in both groups, whereas complete radiographic disappearance of extruded material occurred only with the bioceramic sealer [11].

This study has several limitations. Only one sealer type was tested, no conventional sealer was included as a comparator, and the follow-up period was limited to 28 days. Anatomical heterogeneity among extracted teeth may also have contributed to baseline differences in AOC and AE despite random allocation. Two-dimensional radiography could not quantify material mass or volume, and the traced area may have been influenced by small variations in projection geometry, segmentation, material redistribution, and overlap with the gutta-percha cone. These sources of variability were minimized by standardized radiographic positioning and exposure parameters, calibration of each image, and consistent application of the same 6 × 10-mm region of interest at all time-points. The findings should therefore be interpreted as changes in projected radiopaque area rather than as direct evidence of sealer expansion, dissolution, resorption, or biological integration.

Future studies should include adequately powered comparisons with other sealers, longer observation periods, and three-dimensional micro-computed tomography or surface scanning combined with registered subtraction radiography to distinguish changes in volume from changes in projection.

## Conclusions

Intracanal radiopaque area remained stable over 28 days, whereas area of extrusion showed different modification over time depending on different experimental condition. The greatest mean increase occurred in the dry, sponge-supported group. Moisture and periapical status may therefore affect the radiographic appearance of extruded NeoSealer Flo.

## Data Availability

The specimen-level dataset and statistical output are available from the corresponding author on reasonable request.
